# With or Without You: Altered Plant Response to Boron-Deficiency in Hydroponically Grown Grapevines Infected by Grapevine Pinot Gris Virus Suggests a Relation Between Grapevine Leaf Mottling and Deformation Symptom Occurrence and Boron Plant Availability

**DOI:** 10.3389/fpls.2020.00226

**Published:** 2020-03-03

**Authors:** Sara Buoso, Laura Pagliari, Rita Musetti, Flavio Fornasier, Marta Martini, Alberto Loschi, Maria Chiara Fontanella, Paolo Ermacora

**Affiliations:** ^1^Department of Agricultural, Food, Environmental and Animal Sciences, University of Udine, Udine, Italy; ^2^CREA Research Centre for Viticulture and Enology, Gorizia, Italy; ^3^Department for Sustainable Process, Agricultural Faculty, Università Cattolica del Sacro Cuore, Piacenza, Italy

**Keywords:** boron deficiency, grapevine leaf mottling and deformation, grapevine pinot gris virus, boron transporters, symptom expression

## Abstract

Despite the increasing spread of Grapevine Leaf Mottling and Deformation (GLMD) worldwide, little is known about its etiology. After identification of grapevine Pinot gris virus (GPGV) as the presumptive causal agent of the disease in 2015, various publications have evaluated GPGV involvement in GLMD. Nevertheless, there are only partial clues to explain the presence of GPGV in both symptomatic and asymptomatic grapevines and the mechanisms that trigger symptom development, and so a consideration of new factors is required. Given the similarities between GLMD and boron (B)-deficiency symptoms in grapevine plants, we posited that GPGV interferes in B homeostasis. By using a hydroponic system to control B availability, we investigated the effects of different B supplies on grapevine phenotype and those of GPGV infection on B acquisition and translocation machinery, by means of microscopy, ionomic and gene expression analyses in both roots and leaves. The transcription of the genes regulating B homeostasis was unaffected by the presence of GPGV alone, but was severely altered in plants exposed to both GPGV infection and B-deficiency, allowing us to speculate that the capricious and patchy occurrence of GLMD symptoms in the field may not be related solely to GPGV, but to GPGV interference in plant responses to different B availabilities. This hypothesis found preliminary positive confirmations in analyses on field-grown plants.

## Introduction

In the last 15 years, vineyards in northeast Italy have been affected by a new disease, called Grapevine Leaf Mottling and Deformation (GLMD), because of the symptoms it causes on grapevine plants (Martelli, 2014). GLMD has been associated with the presence of Grapevine Pinot Gris virus (GPGV), a virus firstly identified in vineyards in Trentino (North Italy) (Giampetruzzi et al., 2012) and now reported to be distributed in Europe (Glasa et al., 2014; Mavrič Pleško et al., 2014; Beuve et al., 2015; Gazel et al., 2016; Eichmeier et al., 2017), East Asia (Cho et al., 2013; Rasool et al., 2017), Australia (Wu and Habili, 2017) and North (Al Rwahnih et al., 2016; Poojari et al., 2016) and South America (Fajardo et al., 2017; Zamorano et al., 2019). GPGV is a positive-sense single-stranded RNA-virus belonging to the Betaflexiviridae virus family, genus Trichovirus (Martelli, 2014). It is localized in the bundle sheath cells (BSCs) of the phloem parenchyma (Tarquini et al., 2018) and it is probably spread by the feeding activity of the grapevine eriophyoid mite *Colomerus vitis* (Malagnini et al., 2016).

Symptoms associated with GPGV infection include delayed budburst, stunted shoots, leaf distortion and mottling, increased berry acidity and poor yield (Saldarelli et al., 2015; AWRI, 2018). Symptoms are most distinct at the beginning of the season and are less apparent on late season growth, with infected plants reported to produce GLMD symptomless shoots and leaves from June onward (Bertazzon et al., 2017). The association between the symptomatology and the presence of the virus is unclear, with several reports documenting a great number of symptomless grapevines hosting GPGV (Giampetruzzi et al., 2012; Saldarelli et al., 2013, 2015; Forte et al., 2014; Mavrič Pleško et al., 2014; Bianchi et al., 2015). Furthermore, transmission electron microscopy (TEM) investigations performed on infected tissues of field-grown grapevines have not revealed any differences in ultrastructural cytopathy induced by GPGV in symptomatic or asymptomatic plants (Tarquini et al., 2018).

Studies on the molecular variability of the virus have shown that genetically distinct GPGV isolates exist and can be roughly grouped into “lineages” matching symptom expression. While Saldarelli et al. (2015) proposed a two-clade-classification, Bertazzon et al. (2017) classified GPGV isolates into three groups: clade A, mainly represented by isolates originating from symptomless grapevines, and clades B and C, mostly containing isolates from plants with symptoms. This clustering partially matched Saldarelli’s two-clade-classification (Saldarelli et al., 2015), with the difference being that the “symptomatic” clade B proposed by Bertazzon et al. (2017) is included in Saldarelli’s asymptomatic clade. The absence of a strict and complete correspondence between GPGV isolate clustering and symptom expression is in line with the strong similarity of the genome sequences of GPGV isolates collected from symptomatic and asymptomatic grapevines reported in various studies (Poojari et al., 2016; Reynard et al., 2016; Al Rwahnih et al., 2016). The three-clade classification proposed by Bertazzon et al. (2017) has been recently partially supported by the phylogenetic analysis of various full-length GPGV genomes (Tarquini et al., 2019a). Even though no conclusive evidence of an association of specific symptoms with a particular molecular variant of the virus was provided, a possible difference in cell-to-cell trafficking and systemic spread by different GPGV isolates within infected tissues was hypothesized (Tarquini et al., 2019a). A further interpretation of the unclear association between the symptomatology and GPGV presence has been suggested by Bianchi et al. (2015), who proposed a correlation between symptom expression and virus titer, with a higher value found in symptomatic plants. Bertazzon et al. (2017) implemented this hypothesis concluding that both genetic variant and viral titer can be correlated with disease manifestation. Recently, GPGV agroinoculation in *Nicotiana benthamiana* and grapevine plants demonstrated that both “symptomatic” and “asymtpomatic” GPGV clones induced visible symptoms in the plant hosts as well as identical ultrastructural modifications (Tarquini et al., 2019b). Differences between the titer of the two GPGV clones in leaves distant from the inoculation point led the authors to speculate that the two clones may have a different ability to move systemically, in line with strain-specific polymorphisms in the movement protein sequence reported by Saldarelli et al. (2015) and Tarquini et al. (2019a). Thus, although the various above-mentioned investigations pointed toward GPGV involvement in GLMD, the mechanism(s) by which the symptom development is triggered in the grapevines is (are) still unclear, allowing consideration of new factors.

Interestingly, the different epidemiological traits of GLMD hint at alterations in plant boron (B) nutrition. Among them, the capricious and discontinuous nature of GLMD symptoms during the growing season and between years, and variations in the type of alterations caused to leaf and shoot morphology in GLMD affected grapevines in the field (such as interveinal chlorosis of the older leaves, cupping and malformation of the young leaves, shortening of the apical internodes) reminded us of the typical B deficiency phenotype in grapevine (Scott and Schrader, 1947; Christensen et al., 2006; Osler et al., 2016; Ermacora et al., 2017). Plant roots take up B primarily as boric acid (B(OH)_3_), a small, uncharged molecule that is relatively permeable across biological membranes (Dordas and Brown, 2000; Stangoulis et al., 2001). Despite the relatively high abundance of boric acid in soil, its availability can be affected by several factors including pH, soil texture, climatic conditions such as rainfall and temperature (Shorrocks, 1997; Yan et al., 2006) and also by antagonistic effects from high levels of nitrogen, potassium, calcium and organic matter content (Goldberg, 1997). This extreme susceptibility to the environmental conditions explains the high prevalence of B deficiency and toxicity affecting different crops throughout the world and across a wide range of climates and soil types (Shorrocks, 1997; Argust, 1998; Park and Schlesinger, 2002). A low B availability for the plant implies strong impairment in plant metabolism (Camacho-Cristóbal et al., 2002, 2004; Han et al., 2008) and, in agricultural systems, a huge impact on plant productivity by reducing not only yield quantity but also quality (Loomis and Durst, 1992; Shorrocks, 1997). A correlation between viral-disease symptom expression and plant B homeostasis has already been described in other pathosystems, underlining how B supply leads to viral symptom remission or alleviation (Cooper et al., 1976; Shimomura, 1982; Bengsch et al., 1989). Moreover, Lim et al. (2014) demonstrated how the coat protein of Alternanthera mosaic virus interacts with the host B transporter protein NbBOR1 in *N. benthamiana*, enabling speculation about a direct interaction between viruses and B transporters.

In this work, we investigate the role of B in GLMD symptom expression and the interactions occurring in the tripartite B-GPGV-plant system. By exploring the presumptive GPGV interference in B homeostasis, B content was examined in root and leaf tissues upon B deficiency, imposed both on healthy and virus-infected plants. The effects on B acquisition and translocation machinery were explored by means of expression analysis of key genes such as B transporters in roots and leaves. Our data, based on investigations on hydroponically grown grapevines, are consistent with a model where GPGV infection suppresses the plant response to B starvation, enhancing the B shortage effect and leading to GLMD symptom occurrence. Preliminary positive confirmations by the analyses on some field-grown plants demand for further in-field surveys focusing on B homeostasis as a new actor in GPGV-grapevine interaction.

## Materials and Methods

### Plant Material

To investigate the roles of GPGV and B in GLMD symptom expression, a total of 32 healthy and GPGV-infected *Vitis vinifera* cuttings obtained by rooting in sterile perlite were grown in a hydroponic system, with B-sufficient or B-deficient nutrient solution. Cuttings were obtained from dormant canes of grapevines (cv Pinot gris, clone VCR 5), collected in December 2017 from symptomatic and asymptomatic plants of a 12-year-old vineyard located in Farra d’Isonzo (Gorizia Province, Italy; 45.91676765918134 N, 13.530162621173078 E). The eventual occurrence of the main grapevine viruses (Giampetruzzi et al., 2012; Saldarelli et al., 2015; Bertazzon et al., 2017; Gentili et al., 2017; Morán et al., 2018; Tarquini et al., 2018; Marra et al., 2019; Tarquini et al., 2019a, b) was checked as described in Tarquini et al. (2018) and Tarquini et al. (2019a). The presence of viruses included in the Italian certification program (Bertazzon et al., 2002) was excluded, while the detection of viruses and viroids reported in Pinot gris tissues simultaneously with GPGV (Giampetruzzi et al., 2012 and Saldarelli et al., 2015, i.e. grapevine rupestris stem pitting-associated virus - GRSPaV-, hop stunt viroid - HSVd-, grapevine yellow speckle viroid 1 and 2 -GYSVd-1 and GYSVd-2- grapevine rupestris vein feathering virus -GRVFV- and grapevine Syrah virus 1-GSyV-1-) revealed the ubiquitous presence of GRSPaV, HSVd and GYSVd-1.

Since every cane collected in the field was positive to GPGV, eight GPGV-free Pinot gris (clone VCR 5) canes were provided by the Phytosanitary service of Friuli Venezia Giulia (ERSA) and tested as described above. Also in this case, GRSPaV, HSVd, and GYSVd-1 were found to be ubiquitous and no other virus was detected. Canes were subsequently divided into two homogeneous 20-cm-long cuttings and distributed equally between the two different nutritional conditions to obtain eight healthy self-rooted plants growing in B-sufficient medium (hereafter named GPGV−/+B) and eight healthy self-rooted plants growing in B-deficient medium (hereafter named GPGV−/−B). GPGV-positive canes were selected from among those collected in the field according to the GPGV variants present, determined as described in the “Sequencing of GPGV Variants” section, to obtain four canes infected with GPGV isolates belonging to the “symptomless” clade A and four canes infected with GPGV isolates belonging to the “symptomatic” clade C (Bertazzon et al., 2017). These eight dormant GPGV-infected canes were divided into two sets of 20-cm-long cuttings and distributed equally between +B and −B conditions to obtain eight infected self-rooted plants growing in B-sufficient medium (hereafter named GPGV+/+B) and eight infected self-rooted plants growing in B-deficient medium (hereafter named GPGV+/−B) (Supplementary Table 1).

Starting from the end of April, plants were transferred to hydroponic conditions and maintained in a greenhouse with temperatures and photoperiod replicating typical spring to early summer field conditions. Hoagland medium was used as the nutrient solution (Hoagland and Arnon, 1950) and was prepared with distilled water purified by a column with Amberlite IRA 743^®^ B-chelating resin (Darwish et al., 2015). This B-deprived nutrient solution was used to grow the plants in B deficiency conditions(−B). To prevent B contamination, all components of the hydroponic system were of plastic material and the filters mounted in the recirculating solution system were supplemented with Amberlite IRA 743^®^ in the −B experimental set. For the full nutrient solution conditions (+B), 0.5 ppm of B supplemented as B(OH)_3_, were added to the nutrient solution. The B content in +B and −B nutrient solution was monitored with an ICP-MS (Inductively Coupled Plasma-Mass Spectrometry; Nexion 350, Perkin Elmer, Waltham, MA, United States). The nutrient solutions were replaced weekly.

Plants were constantly monitored for macroscopic symptoms expression. After 100 days of hydroponic growth, macroscopic symptoms were evaluated and samples were collected for the different analyses. The following biometric parameters were determined: dry canopy weight (shoot plus leaves); dry root weight; internode length; and number of leaves. While symptom evaluation and biometric parameter measurements involved eight plants *per* experimental condition, microscopy observations, identification of GPGV isolate variants and virus relative titer quantification, nutrient content analysis and gene expression investigation were performed on five plants *per* experimental condition.

Regarding the field investigation, leaves from asymptomatic and symptomatic vines of cv Pinot gris were collected in May 2019 from the experimental fields of Farra d’Isonzo (Gorizia, Italy). The leaves of symptomatic and asymptomatic plants were sampled and the phytosanitary status of the plants was verified as described above, with GPGV detected in each sample confirming the massive presence of GPGV in vineyards of our region (Bianchi, personal communication) (Supplementary Table 1). For GPGV variant identification, relative virus titer quantification and gene expression analysis, the leaves of six asymptomatic and six symptomatic plants were chosen, including the plants from which the original canes were collected in 2017 for the hydroponics experiments.

### Light and Transmission Electron Microscopy

For root observations, plants were carefully removed from the pots, nutrient solution was washed from the roots and a 5 mm-long portion of the fine roots corresponding to the secondary growth area (Gambetta et al., 2013) were cut, fixed in 3% glutaraldehyde and processed as described in Tarquini et al. (2018). Leaf segments (3–4 mm in length) including both vein tissues and surrounding parenchyma cells were fixed in 3% glutaraldehyde and processed in the same manner. To compare root and leaf midrib histology in the different experimental conditions, semi-thin sections (1 μm in thickness) of resin-embedded material, prepared as described above, were cut using an ultramicrotome (Reichert Leica Ultracut E ultramicrotome), stained with 1% toluidine blue, and examined using a Zeiss Axio Observer Z1 microscope (Carl Zeiss GmbH, Munich, Germany). Five samples per condition were examined and from each sample at least ten non-serial cross-sections were observed. Images were acquired with a 20× objective and stitched using the “grid stitching” plugin (Preibisch et al., 2009) of ImageJ version 1.49m software (National Institutes of Health, Bethesda, MD, United States). Ultrathin sections (60–70 nm) were cut using an ultramicrotome (Leica Reichert Ultracut E ultramicrotome, Leica Microsystems, Wetzlar, Germany) and collected on 200 mesh uncoated copper grids. Sections were then stained with UAR-EMS (uranyl acetate replacement stain) (Electron Microscopy Sciences, Fort Washington, PA, United States) and observed under a PHILIPS CM 10 TEM (FEI, Eindhoven, The Netherlands), operated at 80 kV and equipped with a Megaview G3 CCD camera (EMSIS GmbH, Münster, Germany).

Five plants *per* experimental condition and five non-serial cross-sections from each sample were analyzed.

### Sequencing of GPGV Variants

RNA was extracted from leaf tissues using a Spectrum RNA Kit (Sigma-Aldrich, St. Louis, MO, United States) following the manufacturer’s instructions. Extracted RNAs were DNase treated and reverse transcribed into cDNA with a QuantiTectReverse Transcription Kit (Qiagen GmbH) following the manufacturer’s instructions. Portions of the movement protein (MP) and coat protein (CP) regions were amplified with the DetF/DetR primer pair (Morelli et al., 2014) according to the protocol suggested by Bertazzon et al. (2017). Sequencing was carried out in both directions using automated equipment (Genechron Service, Rome, Italy). The obtained sequences were aligned with reference sequences selected from GenBank (Saldarelli et al., 2015; Bertazzon et al., 2017) using ClustalW (Thompson et al., 1994) with the following parameters: gap opening penalty: 10, gap extension penalty: 0.2, 30% divergent cutoff. 460-bp-long portions of the aligned sequences were used for construction of a Neighbor Joining (NJ) tree (Saitou and Nei, 1987) employing Mega X (Kumar et al., 2018). The reliability of the analyses was subjected to a bootstrap test with 5000 replicates. 12 plants infected with 12 different GPGV strains were selected and used as reported in “Plant Material” section.

### Relative Quantification of the Virus

RNA was extracted from root and leaf tissues using a Spectrum RNA Kit (Sigma-Aldrich, St. Louis, MO, United States) following the manufacturer’s instructions (protocol A for root tissues and protocol B for leaf tissues) and cDNA was prepared as described above. Virus relative quantification in leaf and root tissues of GPGV+plants was carried out by RT-qPCR using the primer pairs GPgV504F/GPgV588R (Bianchi et al., 2015). *VvGAPDH* (glyceraldehyde-3-phosphate dehydrogenase) was chosen for the normalization according to Bertazzon et al. (2017) and Bianchi et al. (2015), using the primer pairs described in Table 1. SsoFast EvaGreen Supermix (Bio-Rad Laboratories Inc., Hercules, CA, United States), cDNA obtained from 5 ng of RNA, and specific primers were combined in a total volume of 10 μl. Every reaction was performed at 95°C for 3 min, 40 cycles of 95°C for 5 s and 58°C for 5 s, followed by a melting curve analysis from 65°C to 95°C to check primer specificity. Relative quantification of the virus in grapevine tissues was calculated with the comparative Cq (*r* = 2^^–ΔΔ*Cq*^) method, using the sample with the smallest amount of the virus as a control (Bertazzon et al., 2017). Statistical analyses were performed by SigmaPlot 12.0 (SigmaPlot Software, CA, United States), using unpaired *t*-tests. Five and six plants *per* experimental condition were analyzed in the hydroponic and in-field investigation, respectively. Three technical repeats were performed for each sample.

**TABLE 1:** List of primers and the accession number of sequences used for housekeeping gene selection.

**Gene**	**NCBI accession no.**	**Forward primer 5′–3′**	**Reverse primer 5′–3′**	**nM**	***M*-value**
*Vv60SRP*	XM_002270599.3	TTCAATGTCGGCACCTCATA	CCTCCGATGTCTCTCTCCAG	300	0.25
*VvUBQCF*	XM_002274299.3	CTATATGCTCGCTGCTGACG ^a^	AGCCAGGCAGAGACAACTC ^a^	300	0.41
*VvEF1-alpha*	XM_002284888.3	TTTGCTGTTCGTGACATCCCG	GCTTCCTCTGTTGAGCTCCC	300	0.31
*VvGAPDH*	XM_002263109.3	GCTGCTGCCCATTTGAAG^b^	CCAACAACGAACATAGGAGCA^b^	300	0.29

*^*a*^Santi et al., 2013. ^*b*^Bianchi et al., 2015.*

### ICP-OES Analysis

Plant material was dried in an oven at 60°C to constant weight then milled with a Retsch mill equipped with a 0.2 screen. A 500 mg quantity of the plant material was put in a 50-mL Digitube (AB Sciex) digestion tube together with 6 mL of 65% HNO_3_ (Carlo Erba, Italy). Tubes were put in a Digiprep apparatus (AB Sciex) set at 95°C for 2 h, and then the volume was brought up to 50 mL using reagent-grade water and filtered with ashless paper (white ribbon, Schleicher and Schuell, Dassel, Germany). Blanks were prepared identically to the samples but omitting plant material. B, calcium (Ca), copper (Cu), iron (Fe), magnesium (Mg), manganese (Mn), sodium (Na), phosphorous (P), potassium (K) and zinc (Zn) contents were determined using an Agilent 5100 ICP-OES (Inductively Coupled Plasma–Optical Emission Spectroscopy) apparatus. Accuracy of analysis was verified by using NIST 1573a reference material. Statistical analyses were performed with SigmaPlot 12.0 (SigmaPlot Software, CA, United States). The nutritional condition (+B and −B) and the phytosanitary status (GPGV− and GPGV+) were used as independent factors in a series of two-way ANOVA, where the dependent variable was each time one of the quantitative measurements, followed by a pairwise multiple comparison procedure using Holm–Sidak method. Data were normalized using logarithm for the following quantitative measurements: Cu content in root, P, B and Na content in leaf. Five plants *per* experimental condition and three technical repeats for each sample were analyzed.

### Phylogenetic Analysis

Currently, four B-deficiency-responsive transporters have been identified: two B efflux transporters (BOR1, BOR2) and two aquaporins (NIP5;1 and NIP6;1), which, even though found in different plant species, have been deeply characterized in Arabidopsis (Takano et al., 2002; Takano et al., 2006; Tanaka et al., 2008; Miwa et al., 2013). For this reason, the amino acid sequences of AtBOR1, AtBOR2, AtNIP5;1 and AtNIP6;1 were used to identify the orthologous proteins in *V. vinifera* with the BLASTP program (Protein Basic Local Alignment Search Tool) at the National Center for Biotechnology Information web site^1^.

To perform an evolutionary analysis of BORs and NIPs, 16 BOR and 15 NIP proteins originating from different organisms were used (Supplementary Tables 2, 3). The multiple sequence alignments were performed using ClustalW software with the following parameters: gap opening penalty: 10, gap extension penalty: 0.2, 30% divergent cutoff. The evolutionary history was inferred using the NJ method (Saitou and Nei, 1987) with the bootstrap test (5000 replicates) in MEGA X software (Kumar et al., 2018).

### Gene Expression Analysis

To evaluate a possible role for GPGV in altering grapevine B uptake and homeostasis, the transcriptional regulation of B transporter genes activated in the plant response to B deficiency was analyzed in five plants for each different experimental condition using real-time experiments performed on a CFX96 instrument (Bio-Rad Laboratories, Richmond, CA, United States). cDNA was prepared as described above. The gene and primer sequences for the expression analysis are reported in Table 2. The reference gene was selected by comparing *Vv60SRP* (60S ribosomal protein), *VvUBQCF* (ubiquitin conjugating factor), *VvEF1-alpha* (elongation factor 1-alpha) and *VvGAPDH* gene expression (Table 1). The gene stability measures (M-values) were calculated according to the geNorm program (Vandesompele et al., 2002; Table 1). The *Vv60SRP* gene was found to be the most stably expressed gene and so the most suitable as a reference gene. SsoFast EvaGreen Supermix (Bio-Rad Laboratories Inc., Hercules, CA, United States), cDNA obtained from 5 ng of RNA, and specific primers were used in a total volume of 10 μl for gene expression in root tissues. Gene expression analyses in leaf tissues were carried out with cDNA from 10 ng of RNA in a total volume of 15 μl. Cycling conditions were the same as described for virus relative quantification. Primers were designed using Primer3 software^2^ and each primer specificity evaluated with the BLASTN (Nucleotide Basic Local Alignment Search Tool) algorithm (Altschul et al., 1997). Primer pair efficiency (E) was evaluated as described by Pfaffl (2001) on the standard curves of different dilutions of pooled cDNA. A mean normalized expression (MNE, Muller et al., 2002) for each gene of interest was calculated by normalizing its mean expression level to the mean expression level of the *Vv60SRP* gene. Three technical repeats and at least five individuals concurred with the gene MNE determination. Statistical analyses were performed with SigmaPlot 12.0 (SigmaPlot Software, CA, United States). In the hydroponic investigation, the nutrition condition (+B and −B) and the phytosanitary status (GPGV− and GPGV+) were used as independent factors in a series of two-way ANOVA, where the dependent variable was each time one of the quantitative measurements, followed by a pairwise multiple comparison procedure using Holm–Sidak’s method. Data were normalized using logarithm for the following quantitative measurements: *VvNIP5* expression in root and *VvNIP5* and *VvNIP6* in leaf. For in-field investigation, statistically significant differences between symptomatic and asymptomatic plants were determined using unpaired *t*-test. Five and six plants *per* experimental condition were analyzed in the hydroponic and in-field investigation, respectively. Three technical repeats were performed for each sample.

**TABLE 2:** List of primers and the accession number of sequences used in real-time PCRs.

**Gene**	**NCBI accession no.**	**Forward primer 5′–3′**	**Reverse primer 5′–3′**	**nM**
*VvBOR1*	XM_002282465.3	CGAAGATACAAGGTGTTGGAGGA	AGACCGTCTGGAACAAGGTG	400
*VvBOR2*	XM_010653992.2	TTGCAATGGAGGGAGAGATGG	ATGCCTAATTTCACCACGGC	400
*VvBOR3*	NM_001280891.1*	CTCAGACAGGGTATGCAGCC	TGCTTCTCCCGTTCTTGGAC	400
*VvNIP5*	XM_002276283.4	GTCCCTTCGGTCAGCATTGG	GCCAATTCCCCCACAGCTC	400
*VvNIP6*	XM_002272952.3	TCCTTCTCATTCAGGGGCGT	ATCCCACTGCTCTGGTGTCG	400

*This primer pair amplifies every gene transcript variant.

## Results

### Symptom Expression and Development in Hydroponically Grown Grapevines

**FIGURE 1:**
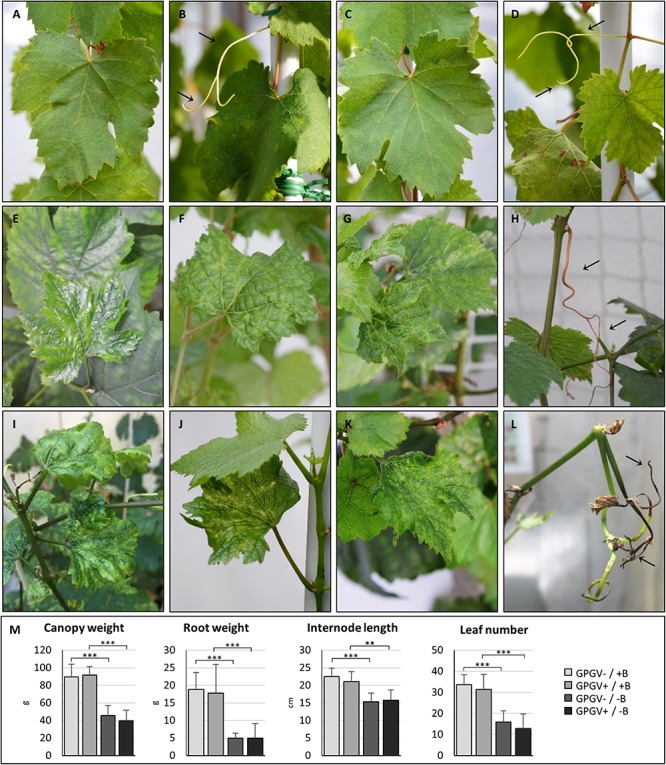
Phenotypes of representative plants at 100 days. Healthy grapevines grown in +B conditions did not express unusual symptoms either in leaves **(A)** or in tendrils **(B)**. No significant difference was observed in the phenotype of GPGV-infected plants grown in +B conditions **(C,D)**. Healthy B-deprived plants showed leaves characterized by a translucent appearance and interveinal chlorosis **(E),** blistering **(F)** and reduced size **(G)**. Other symptoms were tendril necrosis dieback of canes, and tip necrosis **(H)**. Comparable symptoms were observed in GPGV-infected B-deprived grapevines **(I–L)**. B-deficiency, but not the presence of GPGV, strongly affected plant phenotype and was supported by measurements of biometric parameters such as canopy and root weight, internode length and leaf number **(M)**. Results are expressed as mean ± SD (*n* = 8). Statistical significance was determined using two-way ANOVA, followed by Holm-Sidak multiple comparison test (*P ≤ 0.05, **P ≤ 0.01, ***P ≤ 0.001 and ns: no significant difference). *F*- and *P*-values are reported in Supplementary Table 4.

Plants grown in +B conditions did not express disease symptoms throughout the whole trial, and no macroscopic differences were noticeable between healthy (GPGV−/+B; Figures 1A,B) and GPGV-infected (GPGV+/+B; Figures 1C,D) plants. B deficiency symptoms appeared within the 11th and the 13th week after the beginning of the experiment, with no statistically significant differences between healthy and infected plants (unpaired *t*-test, *n* = 8). In GPGV−/−B plants, symptoms in mature leaves included curling, blistering, translucent appearance and interveinal chlorosis, while young symptomatic leaves were smaller than normal, distorted, and had chlorotic spots (Figures 1E–G). Other symptoms were tendril necrosis, dieback of canes and tip necrosis (Figure 1H). Comparable symptoms were observed in GPGV+/−B grapevines (Figures 1I–L). The strong impact of B-deficiency on plant phenotype was also supported by canopy and root fresh weight measurements, internode length measurement and leaf count, all evidencing reduced vegetative growth. As already observed for symptom expression, these biometric parameters were not influenced by the presence of GPGV in either the +B or −B conditions (Figure 1M and Supplementary Table 4).

### Microscopy Observations

**FIGURE 2:**
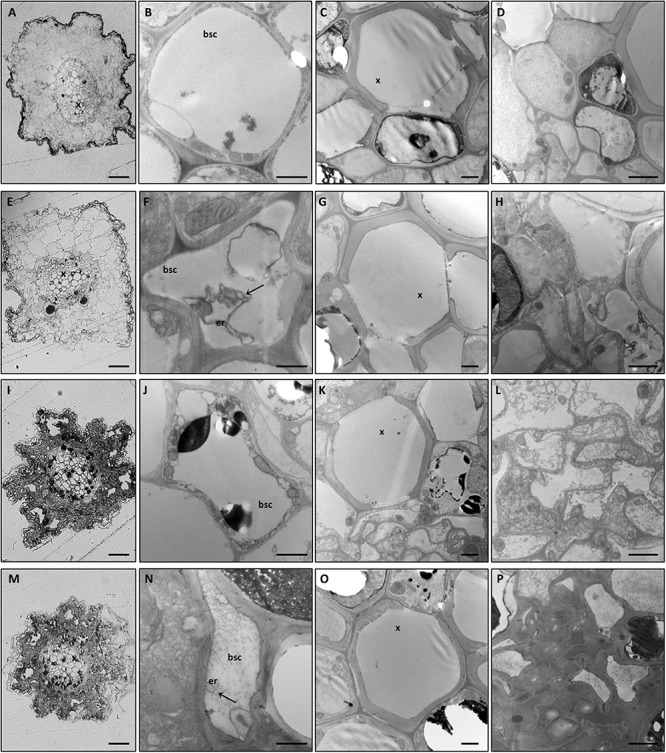
Cytological and ultrastructural organization of roots. Roots of healthy grapevines grown in +B conditions displayed well-preserved histological **(A)** and ultrastructural **(B–D)** organization. GPGV-infected roots grown in +B conditions also presented regular tissue organization **(E)**. Besides the endoplasmic reticulum deformations observed in bundle sheath cells **(F, arrow)**, no other alterations were observed **(G,H)** relative to healthy plants. In B-starvation conditions, roots of healthy plants were affected by strong perturbations of both cortical and vascular regions **(I)**. Despite xylem vessels seeming to be unaffected **(K)**, consistent flavonoid deposits were present in parenchyma cells **(J–K)** and convoluted cell walls and necrosis affected most of the cells in the cortical region **(L)**. GPGV-infected B-deprived roots were affected by the same cytological alterations observed in B-deprived healthy plants **(M)**. Besides putative ER rearrangements **(N)**, no other difference was detected at an ultrastructural level **(O,P)**, when compared to the root tissue organization of healthy plants. For each condition, five different plants were analyzed (*n* = 5), at both the light microscope and transmission electron microscope. bsc: bundle sheath cell, er: endoplasmic reticulum, x: xylem. Bars: A, E, I and P: 50 μm; B, F, J and N: 1000 nm; C, D, G, H, K, L, O and P: 2000 nm.

Alongside the macroscopic evaluations, semi-thin transverse sections of roots and leaf midribs from the different experimental conditions were observed by light (LM) and transmission electron microscopy (TEM). Plant nutritional status dramatically affected root tissue organization. In fact, GPGV−/+B and GPGV+/+B plants displayed well-preserved root cells with normal histological and ultrastructural organization (Figures 2A–H, respectively). GPGV, detected in the BSCs of roots of GPGV+/+B plants, was not associated with any alteration besides membrane-bound organelle, described by Tarquini et al. (2018) as putative endoplasmic reticulum (ER) rearrangement (Figure 2F; Tarquini et al., 2018). In the case of B starvation, GPGV− root tissues displayed strong perturbation of both cortical and vascular regions (Figure 2I). Consistent flavonoid deposits were observed in parenchyma cells (Figures 2J,K) and convoluted cell walls and necrosis affected most of the cells in the cortical region (Figure 2L). Xylem vessel morphology did not seem to be influenced by the altered nutrient conditions (Figure 2K). Similar to the observations in +B conditions, the presence of GPGV was not associated with any significant alteration besides putative ER rearrangement (Figures 2M–P).

**FIGURE 3:**
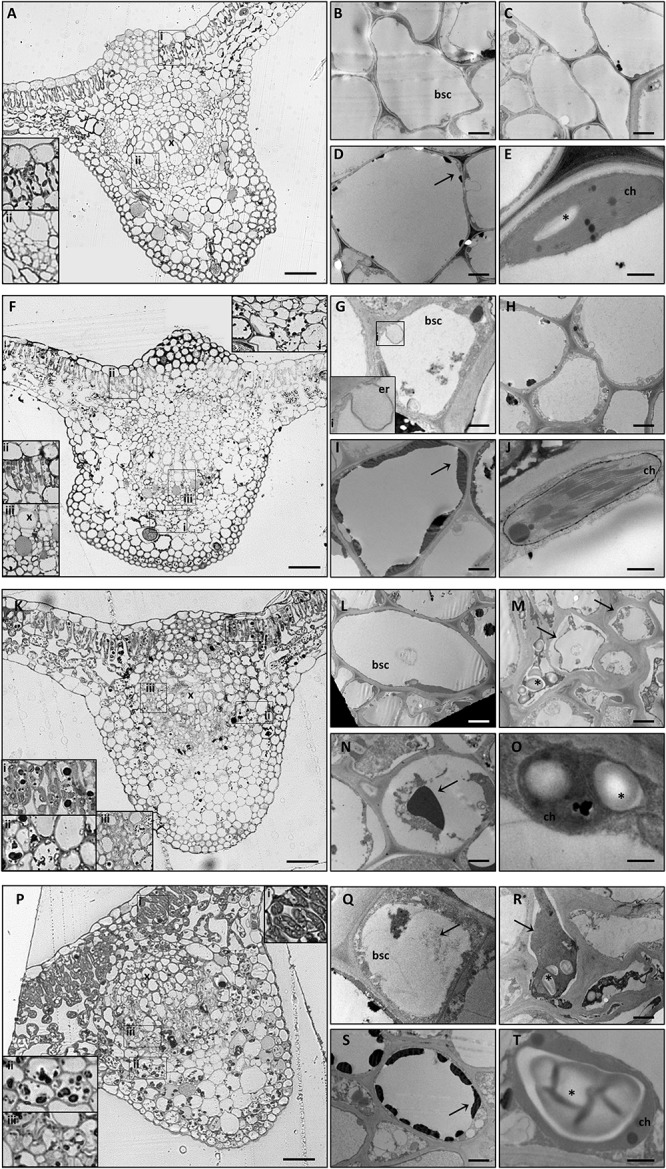
Cytological and ultrastructural organization of the leaf midrib. Healthy grapevines grown in +B conditions did not express specific symptoms at the cytological **(A)** or the ultrastructural **(B–E)** level. GPGV-infected midribs grown in +B conditions presented regular tissue organization **(F)**, both in parenchyma **(F,I,ii)** and in bundle sheath cells **(red arrowheads, F, iii)**. Here, the presence of GPGV was associated with endoplasmic reticulum alterations **(G)**. As in healthy plants **(C)**, cell walls appeared regular and thin **(H)**. Slight flavonoid deposits were observed throughout the midribs **(F,I, arrow)**. No ultrastructural disorganization was characterized in chloroplasts **(J)**. In B-starvation conditions, midribs of healthy plants displayed a regular cytological organization **(K)** and necrosis in different cell types and an increase in flavonoid deposits **(K,i,ii, N, arrow)**. Midrib cells presented plasmolysis **(M,arrows)** and cell wall thickening **(M)**. Chloroplasts were swollen in different cell types due to accumulated starch grains **(O,asterisk)**. In midribs there were no identifiable differences detected at cytological level between B-starved GPGV-infected plants and B-starved healthy plants **(P)**. Virus particles were observed in bundle sheath cells **(Q,arrow)** and cell wall thickenings **(R)**, areas of plasmolysis **(R,arrow)** and flavonoid accumulation **(S,arrow)** and associated with chloroplast alteration due to starch accumulation **(T,asterisk)**. For each condition, five different plants were analyzed (*n* = 5) with both light and transmission electron microscopy. bsc: bundle sheath cell, ch: chloroplast, er: putative endoplasmic reticulum, x: xylem. Bars: A, F, K and P: 50 μm; B, D, G, I, L, N, Q and S: 1000 nm; C, H, M and R. 2000 nm; E, J, O and T: 500 nm.

LM observations of midribs from GPGV−/+B plants identified a regular collateral pattern (Figure 3A) and no cell alteration in the vascular bundles (Figures 3B–E). Besides putative ER modifications associated with the presence of the virus in the BSCs of GPGV+ plants, no other difference between GPGV− and GPGV+ plants grown in full-nutrient solution were detected at the histological (Figure 3F) or ultrastructural levels (Figures 3G–J), although flavonoid accumulation seemed to be more evident following GPGV infection (Figure 3I). Under B starvation, GPGV− plants maintained a regular organization of the vascular tissues (Figure 3K). Nevertheless, midrib cells presented plasmolysis and cell wall thickening, both clearly visible at TEM (Figures 3L–N). Parenchyma cells were also characterized by the presence of massive flavonoid aggregations (Figure 3N). Both LM and TEM observations highlighted chloroplast swelling in different cell types, especially in the palisade parenchyma (Figures 3K,O). Altered chloroplast morphology was caused by the accumulation of starch granules, embedded in the thylakoids (Figure 3O). LM observations of midribs from GPGV+/−B plants (Figure 3P) did not detect any particular difference from the observations in GPGV−/−B plants. Similarly, TEM micrographs of GPGV+/−B midrib cells (Figures 3Q–T) did not reveal any difference when compared to their respective healthy control, with the sole exception of putative ER alterations associated with virus in the BSCs (Figure 3Q).

### Sequencing of GPGV Variants

**FIGURE 4:**
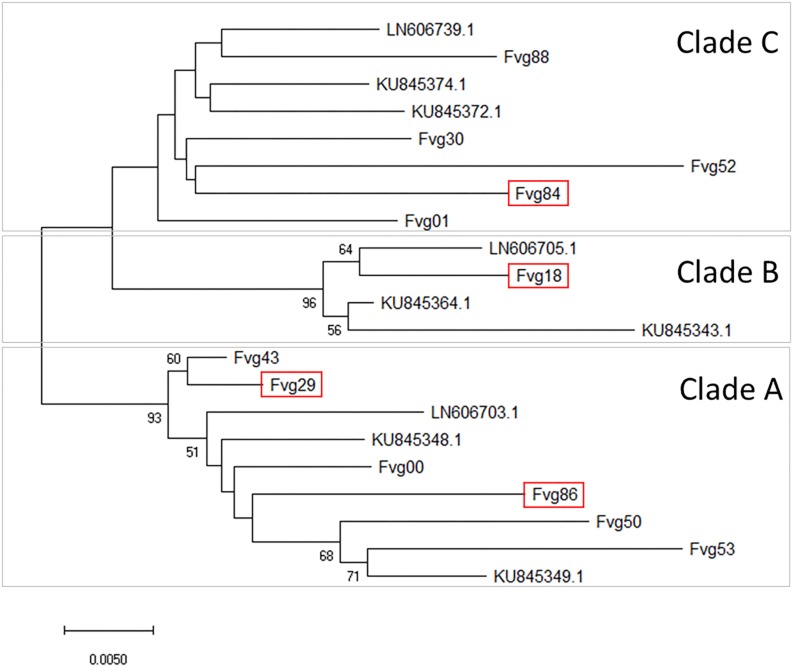
Evolutionary relationships between GPGV isolates. The unrooted Neighbor-Joining phylogenetic tree was obtained with the MEGA X program using the sequences of the DetF/DetR PCR products obtained in this study and selected published sequences considered as references. Three main clades **(A–C)** were separated. For in-field analysis, GPGV isolates with sequences framed in red were added to the isolates originating from canes used for the experiment in controlled conditions. The optimal tree with the sum of branch length = 0.28456690 is shown. The percentage of replicate trees in which the associated taxa clustered together in the bootstrap test (5000 replicates) are shown next to the branches. Bootstrap values (>50) are reported at the nodes. The tree is drawn to scale, with branch lengths in the same units as those of the evolutionary distances used to infer the phylogenetic tree. The evolutionary distances were computed using the Maximum Composite Likelihood method and are in the units of the number of base substitutions per site. All ambiguous positions were removed for each sequence pair (pairwise deletion option). There were a total of 461 positions in the final dataset.

Sequences of the MP/CP genomic portion of the GPGV isolates were aligned and compared with three reference sequences representative of the main clades identified by Saldarelli et al. (2015) and six reference sequences representative of the main clades identified by Bertazzon et al. (2017). An NJ cladogram resolved three main clades, named A, B, and C. Isolates Fvg43, Fvg29, Fvg00, Fvg86, Fvg50, and Fvg53 were grouped into Clade A, which included the reference isolate LN606703.1, KU845348.1, and KU845349.1, representative of isolates originating from symptomless plants in Saldarelli et al. (2015) and Bertazzon et al. (2017). Isolate fvg18 was grouped into Clade B, together with LN606705.1, KU845364.1, and KU845343.1, whose presumptive association with symptom expression is not yet clear (Bertazzon et al., 2017). Isolates Fvg88, Fvg30, Fvg52, Fvg84, and Fvg01 clustered into Clade C with the reference isolates LN606739.1, KU845372.1, and KU845374.1, originating from symptomatic plants in Saldarelli et al. (2015) and Bertazzon et al. (2017; Figure 4). The distribution of the different GPGV variants in the two experimental systems (controlled conditions and field conditions) is summarized in Figure 4. Sequences were deposited in GenBank (Accession numbers: MN587102-MN587113).

### Relative Virus Quantification

**FIGURE 5:**
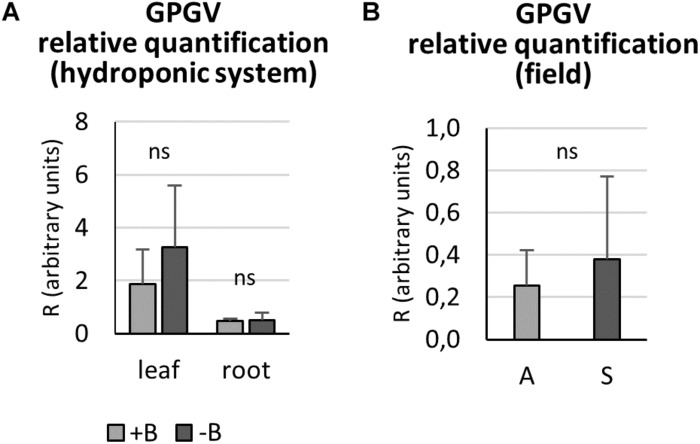
Grapevine Pinot gris virus relative quantification by qPCR. In controlled experimental conditions, both leaf and root tissues of GPGV-infected plants grown in + B solution presented a similar virus concentration when compared to the respective organ of plants grown in B-starvation **(A)**. In the field, GPGV relative quantification in leaves did not lead to any statistically significant differences between asymptomatic and symptomatic plants **(B)**. Results are expressed as mean ± SD (*n* = 5 in controlled experimental condition and *n* = 6 in in-field analysis). Statistical significance was determined using unpaired *t*-test (*P ≤ 0.05, **P ≤ 0.01, ***P ≤ 0.001 and ns: no significant difference). A: asymptomatic plants, S: symptomatic plants.

Relative GPGV titer was quantified in +B and −B GPGV-infected plants grown in controlled experimental conditions, according to Bertazzon et al. (2017). In both leaf and root tissues, nutritional status did not interfere with virus replication capabilities (Figure 5A).

In the field, asymptomatic and symptomatic leaves were sampled and analyzed for virus titer. No statistically significant difference between the two plant groups was detected (Figure 5B), as the means were very similar and the standard deviations were very large.

### Nutrient Content

**FIGURE 6:**
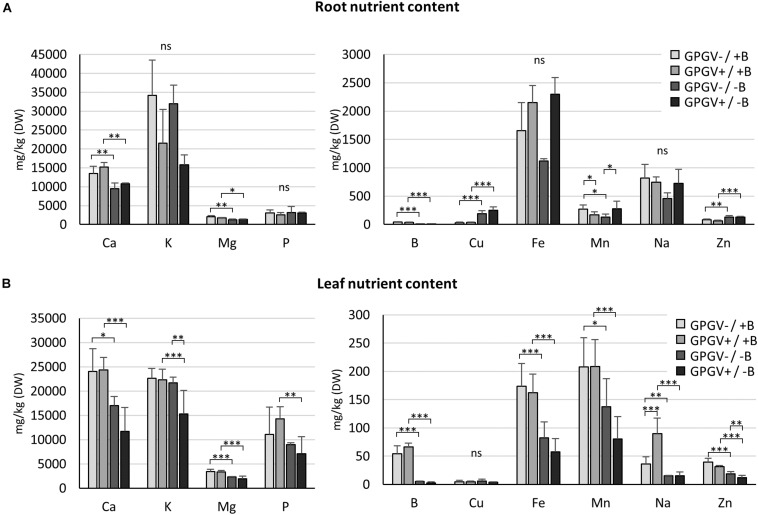
Nutrient concentration in root and leaf tissues by ICP-OES. In root tissues **(A)**, Ca, B, Mg, Cu, and Zn concentration levels were influenced by the sole B starvation conditions. Conversely, GPGV infection altered K, Fe, and Na concentration only in the case of B starvation. In leaf tissues **(B)**, B starvation conditions strongly affected the concentration of some nutrients, such as Ca, Mg, B, and Fe. While K, P, and Mn concentrations were altered only in the case where both stresses were present, Cu levels always remained unaffected and Na increased following infection only in plants grown in the +B solution. The Zn concentration presented a gradual decline throughout the four experimental conditions, being affected both by B deprivation and the presence of GPGV. Results are expressed as mean ± SD (*n* = 5). Statistical significance was determined using two-way ANOVA, followed by Holm–Sidak’s multiple comparison test (*P ≤ 0.05, **P ≤ 0.01, ***P ≤ 0.001 and ns: no significant difference). *F*- and *P*-values are reported in Supplementary Table 5.

To investigate if and how B starvation and GPGV infection interferes with the entire nutrient status of grapevines, the concentration of the main nutrients was quantified in roots and leaves of plants exposed to the different experimental conditions (Figure 6). In root tissues (Figure 6A), B levels fell dramatically by approximately 90% in B deficient plants, regardless of whether GPGV was present or not. Like B, also Ca, Mg, Cu, and Zn concentration levels were influenced only by B factor. B−deprived plants suffered a decrease in the concentrations of Ca (−30% in GPGV− plants and −28% in GPGV+ plants) and Mg (−38% in GPGV− plants and −25% in GPGV+ plants). Conversely, both Cu and Zn levels grew under −B conditions: Cu concentration rose by 527% in GPGV− plants and 560% in GPGV+ plants, while Zn concentration increased by 60% in GPGV− plants and 110% in GPGV+ plants. Furthermore, two-way ANOVA analysis indicated a statistically significant interaction between the two stresses on Mn root content (*P* = 0.005) (Supplementary Table 5). The K, P, Fe, and Na concentrations were not altered by either GPGV infection or B starvation.

In leaf tissues (Figure 6B), the B concentration dramatically dropped by 91 and of 95% in GPGV− and GPGV+plants, respectively, upon B starvation stress induction. B starvation strongly affected the concentration of some nutrients: Ca concentration decreased by 29% in GPGV− and 52% in GPGV+ plants, Mg content was reduced by 32% in GPGV− and 43% in GPGV+ plants, Fe decreased by 53% in GPGV− plants and 65% GPGV+ plants and Mn content was reduced by 34% in GPGV− and 62% in GPGV+ plants, compared to their respective B+ controls. P content was affected only in GPGV+ plants upon B starvation (−50%). Zn concentration was affected both by B deficiency and GPGV infection. Considering B nutrition, GPGV−/−B and GPGV+/−B plants showed, respectively, a decrease of 55% and 62% when compared to their respective +B controls. In double stress condition (GPGV+/−B), a lower Zn content (−34%) was measured in comparison to GPGV−/−B. Two-way ANOVA analysis indicates a statistically significant interaction between the two stresses on K and Na content (*P* = 0.025 and *P* = 0.005, respectively) (Supplementary Table 5). In particular K content decreased by 31 and 29% in GPGV+/−B compared to GPGV+/+B and GPGV−/−B plants. B starvation affected the concentration of Na, with a reduction of 59% in GPGV− and 83% in GPGV+ plants, when compared to their respective B control. In B sufficient condition, GPGV presence induced a rise of 150% in Na content. Cu levels remained unaffected in the different experimental conditions.

### Phylogenetic Analysis

BLASTP analysis identified the *V. vinifera* XP_010652294.1 and NP_001267820.1 proteins as the most similar to AtBOR1, with amino acid sequence identities of 80 and 75%, respectively, and XP_010652294.1 and XP_002282501.1 (VvBOR1; Pérez-Castro et al., 2012) were the most similar to AtBOR2 (sequence identities: 81 and 76%). Moreover, the Phobius program (Käll et al., 2004) predicted that the XP_010652294.1, NP_001267820.1, and XP_002282501.1 proteins contained 10, 10 and 12 transmembrane domains, respectively (Supplementary Table 2), which parallels the 8–12 transmembrane domain proteins that characterize the AtBOR proteins (Wakuta et al., 2015). Regarding proteins homologous to AtNIP5;1 and AtNIP6;1 in *V. vinifera*, we obtained the XP_002276319.1 protein with 81% sequence identity and the XP_002272988.1 protein with 73% sequence identity, respectively.

**FIGURE 7:**
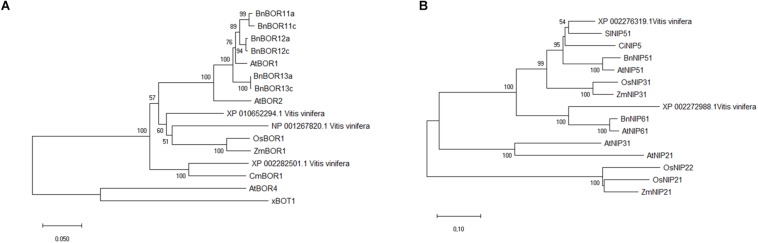
Sequence alignment and phylogenetic analysis of BOR-like and NIP-like proteins. Unrooted phylogenetic tree analysis of putative BOR1-like proteins in different plant species **(A)** showed that putative grapevine BOR1-like proteins clustered with AtBOR1 and other AtBOR1-like proteins and not with AtBOR4. Unrooted phylogenetic tree analysis of putative NIP-like proteins **(B)** in different plant species showed that putative grapevine NIP proteins clustered with the AtNIP5;1 and AtNIP6;1 proteins, belonging to B-channel NIP group II. For construction of both phylogenetic trees, the evolutionary history was inferred using the Neighbor-Joining method. The optimal tree with the sum of branch length = 1.43332686 (BOR) or 2.79539795 (NIP) is shown. The percentage of replicate trees in which the associated taxa clustered together in the bootstrap test (5000 replicates) are shown next to the branches (bootstrap value > 50). The tree is drawn to scale, with branch lengths in the same units as those of the evolutionary distances used to infer the phylogenetic tree. The evolutionary distances were calculated using the Poisson correction method and are in the units of the number of amino acid substitutions per site. This analysis involved 16 amino acid sequences for the BOR phylogenetic tree and 15 amino acid sequences for the NIP phylogenetic tree. All ambiguous positions were removed for each sequence pair (pairwise deletion option). There were a total of 737 positions in the final dataset for the BOR phylogenetic tree and 385 positions for the NIP phylogenetic tree. Evolutionary analyses were conducted in MEGA X. Sequences used for tree construction are reported in Supplementary Tables 2, 3.

To determine the phylogenetic relationships between putative VvBORs and VvNIPs and BOR and NIP proteins belonging to other plant species, phylogenetic trees were constructed using the NJ algorithm of MEGAX software. As shown in Figure 7A, the XP_010652294.1, NP_001267820.1, and XP_002282501.1 proteins grouped in the same clade (*BOR1-like*) with AtBOR1 and AtBOR2. Since XP_002282501.1 was characterized as VvBOR1 (Pérez-Castro et al., 2012), we named XP_010652294.1 as VvBOR2 and NP_001267820.1 as VvBOR3, following the nomenclature of Alva et al. (2015).

The XP_002276319.1 and XP_002272988.1 proteins belong to the NIP group II (Figure 7B), which includes the B-transporting aquaporins (Tanaka and Fujiwara, 2008). In particular, XP_002276319.1 clustered with AtNIP5;1, while XP_002272988.1 clustered with AtNIP6;1. Considering this grouping and the high sequence similarity inferred by BLAST analysis, we decided to name XP_002276319.1 as VvNIP5 and XP_002272988.1 as VvNIP6.

### Gene Expression Analysis

**FIGURE 8:**
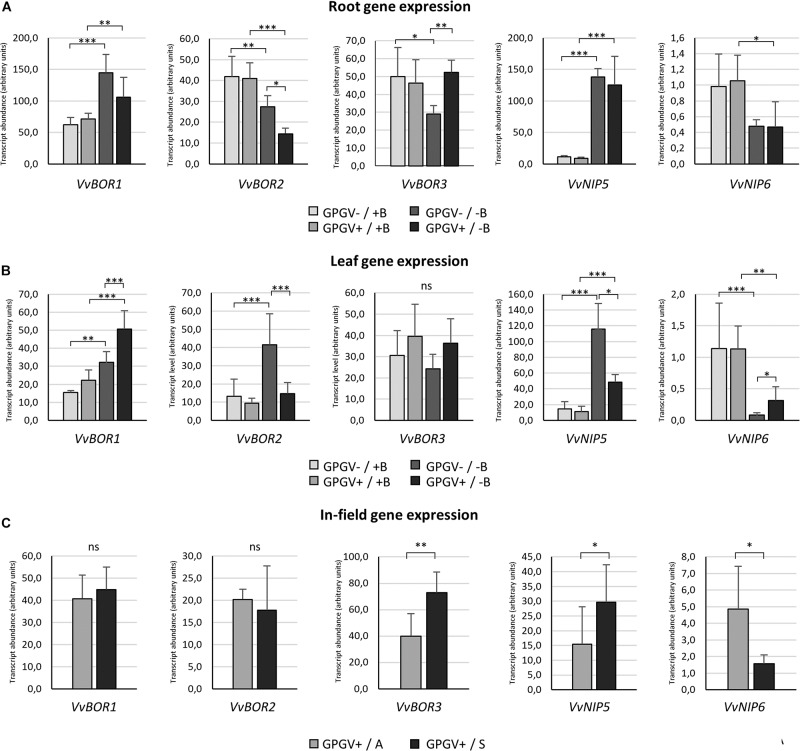
B transporter gene expression in the different experimental conditions. In root tissues **(A)**, B starvation conditions strongly affected the transcript regulation of every gene considered. The presence of GPGV did not interfere with B channel gene expression in + B condition, but altered plant responses to B deprivation in three out the five genes considered (*VvBOR1*, *VvBOR2*, and *VvBOR3*). In leaf tissues **(B)**, plants responded to B starvation with differential regulation of four out the five genes analyzed (*VvBOR1*, *VvBOR2, VvNIP5*, and *VvNIP6*). The expression of these genes was also affected by the presence of GPGV in B-deprived plants alone. As in roots, GPGV interference mostly limited the plants’ attempts to restore B homeostasis. Results are expressed as the mean ± SD (*n* = 5). Statistical significance was determined using two-way ANOVA, followed by Holm–Sidak’s multiple comparison test (*P ≤ 0.05, **P ≤ 0.01, ***P ≤ 0.001 and ns: no significant difference). *F*- and *P*-values are reported in Supplementary Table 6. In plants collected from the vineyard **(C)**, while the expression of the *VvBOR1* and *VvBOR2* genes was not differentially regulated, *VvBOR3* and *VvNIP5* were upregulated in symptomatic plants and *VvNIP6* transcript levels were lower in symptomatic grapevines when compared to asymptomatic ones. Results are expressed as the mean ± SD (*n* = 6). Statistical significance was determined using unpaired *t*-test. *P ≤ 0.05, **P ≤ 0.01, ***P ≤ 0.001, and ns: no significant difference.

The transcriptional regulation of B-deficiency responsive genes was evaluated both in root and in leaf tissues of plants grown in environmentally controlled system. In roots (Figure 8A), B starvation led to an increase in *VvBOR1* transcription (+132% in GPGV−/−B plants when compared to the transcript level observed in GPGV−/+B plants). Although not statistically significant, *VvBOR1* response to B deprivation was reduced by the concomitant presence of the virus (GPGV+/−B) (only +48% compared to GPGV−/+B plants). The transcript abundance of *VvBOR2* was not affected by virus presence in +B condition, being differently regulated only by B availability. Contrary to the observations for *VvBOR1*, *VvBOR2* transcript levels underwent significant reduction (−35%) when GPGV−/+B was compared to GPGV−/−B plants. This decrease was exacerbated by GPGV infection in GPGV+/−B grapevines, where a respective downregulation of 65 and 47% relative to GPGV+/+B and GPGV−/−B plants was observed. Expression of *VvBOR3* was downregulated by B deprivation only in healthy plants, with a decrease of 44% of the transcript abundance between GPGV−/+B and GPGV−/−B grapevines. Virus presence in GPGV+/−B plants led the *VvBOR3* expression to the basal level. Two-way ANOVA analysis confirmed the interaction between B and GPGV factors on *VvBOR3* expression (*P* = 0.022) (Supplementary Table 6). Concerning *VvNIP5*, the only remarkable alteration in its expression was ascribable to B starvation, that in GPGV− plants caused an upregulation of 1145% while in GPGV + plants caused and upregulation of 1289%. In fact, GPGV infection did not affect *VvNIP5* transcriptional regulation in either +B or −B plants. Additionally, when considering *VvNIP6* gene expression, B deprivation altered *VvNIP6* transcript levels only in GPGV+ plants, causing a reduction of −55% in comparison to GPGV+/+B.

In leaves, as observed in roots, B deprivation in healthy plants (GPGV−/−B) caused a 113% increase in *VvBOR1* transcript abundance in comparison to GPGV−/+B plants (Figure 8B). Two-way ANOVA analysis underlined that both B and GPGV factors influenced *VvBOR1* transcript levels (Supplementary Table 6). While GPGV did not change *VvBOR1* transcription in +B conditions, the simultaneous presence of virus and altered nutritional status (GPGV+/−B) resulted in a further upregulation (+367% relative to GPGV−/+B, +127% relative to GPGV+/+B and +56% relative to GPGV−/−B). Expression of the *VvBOR2* gene was upregulated by B starvation only in healthy plants, causing an increase of 208% in GPGV−/−B plants. Similar to *VvBOR1*, *VvBOR2* transcript regulation did not change following virus infection in plants grown in +B conditions. Nevertheless, the concomitant presence of the virus and the altered nutritional status caused a decrease of 66% in GPGV+/−B plants when compared to GPGV−/−B plants, leading the transcript abundance to the basal level. Two-way ANOVA analysis showed the interaction between B and GPGV factors on *VvBOR2* expression (*P* = 0.023). Neither the presence of the virus nor B starvation triggered altered regulation of *VvBOR3* transcription. *VvNIP5* gene expression was upregulated by B deprivation causing a 673% rise in transcript levels in GPGV− plants compared to GPGV−/+B grapevines. The GPGV infection did not alter *VvNIP5* transcription in +B plants, but unlike the data from roots, it dampened the *VvNIP5* response to B starvation, limiting the transcript upregulation observed in healthy plants following B deficiency (only +336% when compared to GPGV−/+B). Finally, *VvNIP6* transcriptional regulation was impaired by B deprivation, with a decrease of 91% in gene expression in GPGV− plants and of 73% in GPGV+ plants. Gene expression was unaffected by the presence of GPGV in plants grown in +B medium. On the contrary, GPGV infection was associated with a 200% rise in transcript levels in GPGV+/B− compared to GPGV−/B− plants. Globally, GPGV altered plant responses to B starvation in three out the five genes considered in roots (*VvBOR1*, *VvBOR2*, and *VvBOR3*) and four out the five genes considered in leaves (*VvBOR1*, *VvBOR2, VvNIP5*, and *VvNIP6*), mostly affecting plant attempts to restore B homeostasis (*VvBOR1* and *VvBOR3* in roots and *VvBOR2*, *VvNIP5*, and *VvNIP6* in leaves).

In plants collected from the vineyard (Figure 8C), gene expression of both *VvBOR1* and *VvBOR2* did not display any differential regulation. *VvBOR3* expression rose by 83% in symptomatic leaf tissue. The *VvNIP5* gene was upregulated in symptomatic plants when compared to asymptomatic ones (+92%). In contrast, *VvNIP6* transcript levels were lower in symptomatic grapevines than in asymptomatic grapevines (−67%). Therefore, regulation of *VvNIP5* and *VvNIP6* in the field paralleled the hydroponic experiments: asymptomatic plants were similar to GPGV+/+B plants and symptomatic plants were similar to GPGV+/−B plants.

## Discussion

### GPGV-Infected Plants Display GLMD-Like Symptoms According to the Nutrient Conditions

Results from different investigations have pointed toward the involvement of GPGV in GLMD (Bertazzon et al., 2017; Tarquini et al., 2019b). Nevertheless, the mechanisms prompting GLMD symptom expression are still unclear. Since GMLD symptoms on grapevine resemble those induced by B-deficiency (Scott and Schrader, 1947; Christensen et al., 2006; Ermacora et al., 2017), we set up an experimental system where healthy and GPGV-infected Pinot gris grapevines were grown in controlled environmental conditions, with different B supplies (+B and −B). Both healthy and GPGV-infected plants grown in full nutrient conditions did not display any symptom until the end of the experiment. Possible causes of the absence of GLMD symptoms could rely on both the difficulty to reproduce disease symptom in controlled conditions (Bos, 1970; Paudel and Sanfacon, 2018) and the adequate B supply through the whole vegetative season. On the other hand, independent of their phytosanitary status, plants grown under B-starvation displayed very similar symptoms to those described for GLMD-affected grapevines in the field, suggesting that alterations in B bioavailability, may coincide with GLMD symptom expression. Symptom similarity at macroscopical level is not confirmed at ultrastructural level. In fact, if GPGV did not significantly alter host histological and ultrastructural organization (beside the presence of ER-like deformed structures, see Tarquini et al., 2018, 2019b), conversely, B starvation led to profound changes at both histological and ultrastructural level (Ermacora et al., 2017). Ultrastructure of GPGV+/−B plants showed the characteristic features of both stresses. Such a compromised organization, never described in field-grown GLMD symptomatic grapevines, is probably caused by strong B starvation. More generally, the lack of a complete correspondence between the macroscopic and microscopic modifications recorded in our experimental system and in the GLMD-affected plants in vineyards is likely due to a mismatch between the complete absence of B in the environmentally controlled conditions and the presumptive B deficiency affecting symptomatic grapevines in the field. In fact, the level of B privation in plants grown in the open field cannot be assumed to be as drastic as in our experiment. Nevertheless, this choice of approach was necessary given the novelty of the topic.

### Neither Genetic Variability nor the Titer of GPGV Interfered With GLMD Symptom Expression

Since both the variant of the virus isolate and the virus titer have been currently supposed to be associated with GMLD symptom occurrence (Saldarelli et al., 2015; Bertazzon et al., 2017), we evaluated the impact of these factors in our experimental system. Concerning genetic variability, the set-up of the experiment led to the formation of two identical groups placed in the two different nutrient conditions (+B and −B) with different GPGV isolates belonging to the “symptomless” clade A and “symptomatic” clade C (Bertazzon et al., 2017). Relative quantification of the viral titer, both in leaves and roots, did not result in significant difference between the two plant groups in different nutrient conditions. Thus, at least in our experimental system, neither genetic variability nor virus titer seem to be associated with GLMD symptom expression.

### Effect of B Deficiency and GPGV Infection on Nutrient Contents

To verify a possible interference of GPGV in nutrient homeostasis, the concentration of the main macronutrients and micronutrients was measured in root and leaf tissues of grapevines in the different experimental conditions. Globally, the concentration of most of the nutrients analyzed was affected by B-deficiency rather than the presence of GPGV, and no overlapping pattern was observed in the plant response to either of the stresses. In the literature, information on the relationships between B-related growth conditions and the homeostasis of the other nutrients is lacking (Tariq and Mott, 2007). In fact, conflicting results have been obtained using different crop species and growth techniques, and also analyzing various plant parts at different growth stages (Lombin and Bates, 1982; Singh and Singh, 1984; Carpena and Carpena-Ruiz, 1987; Mozafar, 1989; Agbenin et al., 1990; Zhou et al., 2014; Sarafi et al., 2018). Unfortunately, to our knowledge, nutrient content measurements have never been carried out in grapevines under B deprivation. In this work, the Ca content decreased in B-deficient plants, independent of the phytosanitary status, both in roots and in leaves, and this was probably due to inhibited translocation to the upper leaves, which has been demonstrated previously in B-deficient tomato (Yamauchi et al., 1986; Ramon et al., 1990). Even though the relationship between B and Ca is still being discussed (Bolaños et al., 2004) and the results about Ca content alteration in B-deficient plants are conflicting, B and Ca share some common functions in plants, playing an important role in cell wall metabolism and the auxin transport process (Dela Fuente et al., 1986). Therefore, it is not surprising that the Ca content decreased under B deficiency. In parallel with Ca and B, the Mg concentration has also been shown to decline in both root and leaf tissues, as reported in cotton and citrus under B deficiency (Zhu et al., 2001; Zhou et al., 2014). A role for B in the physiological process controlling the uptake and transport of Fe and Zn suggested by Dave and Kannan (1981) could explain the alteration of Fe and Zn content observed in roots or/and leaves. Moreover, Zn mitigation of B deficiency has been reported in orange (Swietlik and LaDuke, 1991) and in pistachio (Tavallali, 2017).

Little is known about the effects of virus infection on nutrient uptake, distribution and accumulation in plant tissues, although mineral nutrition plays an important role in plant-pathogen interactions (Datnoff et al., 2007; Huber et al., 2012) and in disease control practices (Dordas, 2008; Gupta et al., 2017). Previous work has demonstrated that the presence of virus affected P, Mg, B, Cu, and Mo content in infected leaves (Overholt et al., 2009; Adkins et al., 2013), but the alterations seemed to vary according to the virus considered. In this work, GPGV did not influence the concentrations of B or those of most nutrients. In fact, following infection, only Na concentration increased in leaves of plants grown in full nutrient solution and the phenotype of grapevines grown in +B did not reveal Na toxicity conditions, suggesting that plant physiology was not significantly impacted. Interestingly, the contents of some nutrients (Mn in roots and K, Na, and Zn in leaves) were affected by the concomitant presence of both stresses, suggesting that viruses interfere in the homeostasis of some elements only when plants face multiple stresses.

### GPGV Altered the B Deficiency Response Both in Leaves and Roots

Given the strong similarity between symptoms displayed by hydroponically grown B-deprived grapevines and those described for GLMD-affected grapevines, we posited a possible viral interference in B homeostasis. To assess whether GPGV infection altered plant B uptake and translocation, gene expression of the main transporters and channels involved in plant responses to B deficiency was analyzed both in roots and leaves (Miwa and Fujiwara, 2010; Reid, 2014; Yoshinari and Takano, 2017).

Even though the passive diffusion of boric acid is considered to satisfy plant demand for B when available in sufficient quantities (Brown et al., 2002), plants have evolved specific boric acid transporters to face limited B availability (Takano et al., 2008). BOR proteins can be classified into two clades that function differently, depending on the B concentration in soil: *BOR1-like* are responsible for B uptake under low B conditions and *BOR4-like* confer tolerance to high B (Wakuta et al., 2015). The borate exporters BOR1 and BOR2 play a key role in B mobilization and distribution in B-deficient conditions: in Arabidopsis, AtBOR1 is involved in xylem loading of B against a concentration gradient in mature endodermal cells and also in all cells of the root tip (Takano et al., 2002; Takano et al., 2008), while *AtBOR2* is expressed in the epidermal cells but not in the endodermis, thus complementing the distribution of AtBOR1 (Miwa et al., 2013). Although localization in the shoot is unclear, AtBOR1 has been suggested as also being involved in B distribution within shoots, possibly by directing B from the xylem to phloem for the preferential supply to young leaves (Takano et al., 2001). No information is available about a possible role for AtBOR2 in leaf tissues. Different BOR transporters have been characterized in several plant species (Nakagawa et al., 2007; Cañon et al., 2013; Leaungthitikanchana et al., 2013; Chatterjee et al., 2014; Zhang et al., 2017), including VvBOR1 in grapevine (Pérez-Castro et al., 2012). In this work, the expression of *VvBOR1*, *VvBOR2*, and *VvBOR3* (orthologous to AtBOR1 and AtBOR2 and grouping in the *BOR1-like* cluster) was analyzed, mostly resulting differently regulated under B-deficient conditions both in root and leaf tissues (with in an increase of *VvBOR1* in a decrease in *VvBOR2* and *VvBOR3* transcript abundance in roots and an increase in *VvBOR1* and *VvBOR2* expression levels in leaves). In Arabidopsis and rice, the accumulation of *AtBOR1* and *OsBOR1* transcripts in leaves and roots was not strongly affected by B limitation, but the BOR1 protein accumulated in the plasma membrane (Takano et al., 2005; Nakagawa et al., 2007). Recently, *BOR1-like* genes were identified in rape and wheat, and different expression patterns of *BOR1-like* genes have been found, probably aimed at different physiological roles in different plant species (Sun et al., 2012; Leaungthitikanchana et al., 2013; Diehn et al., 2019).

The efficient B uptake and allocation under B-limiting conditions are also guaranteed by the AtNIP5;1 and AtNIP6;1 proteins. The NIP family is classified into three sub-groups based on the sequence of the aromatic/arginine (ar/R) constriction region, which determines their specificity for transport substrates (Wallace and Roberts, 2004; Mitani et al., 2008). Respectively, AtNIP5;1 and AtNIP6;1 facilitate B influx into root cells and distribution of B into young growing leaf tissues (Takano et al., 2006; Tanaka et al., 2008). The gene expression of their orthologs in grapevine (*VvNIP5* and *VvNIP6*), both of which cluster in the B-transporting NIP sub-group, was differently regulated in response to B starvation. In fact, an increase in *VvNIP5* and a decrease in *VvNIP6* transcript levels were observed in both roots and leaves. The tolerance to B starvation that characterizes plants overexpressing *AtNIP5;1* (Kato et al., 2008) and the induction of *NIP5;1* expression in roots and/or leaves that have been reported for different models and crop plants subjected to B deficiency (An et al., 2012; Hanaoka et al., 2014; Di Gioia et al., 2017; Diehn et al., 2019), are in line with the transcriptional upregulation of *VvNIP5;1* observed in our experimental conditions. Indeed, *VvNIP5* may act as a boric acid channel essential for plant acclimation to B limitation. On the other hand, the upregulation of *AtNIP6;1* transcript levels (Tanaka et al., 2008) does not reflect the down-regulation of *VvNIP6* following B starvation. Nevertheless, in rape, no up-regulation of *BnNIP6* genes was detected under B limiting conditions in any of the examined tissues (Diehn et al., 2019), confirming a possible different role for NIP6 depending on the plant species. Considering the species specificity that seems to characterize B transporters, and the fact that, to our knowledge, almost no data are available about their role in grapevine upon B deprivation (Pérez-Castro et al., 2012), our results contribute toward understanding grapevine responses to B starvation stress.

Regarding the presumptive interference of GPGV in B homeostasis, the virus did not alter B transporter transcriptional regulation in plants grown in full-nutrient conditions. Conversely, GPGV affected the expression of *VvBOR1*, *VvBOR2*, and *VvBOR3* in roots and *VvBOR1*, *VvBOR2*, *VvNIP5*, and *VvNIP6* in leaves of plants facing B-limited growth conditions. While it is not possible to establish the specific nature of this interaction, our findings indicated an attenuation of B deficiency responses in B-starved GPGV-infected plants.

This conclusion may apparently be in contradiction with both the absence of symptom exacerbation in GPGV+/−B plants (in comparison to GPGV−/B− plants) and the similar B concentration detected by ICP-OES in –B plants, regardless of GPGV presence. In our experimental setting (i.e. optimum B level in +B nutrient solution and total absence of B in -B nutrient solution), the upregulation of B transporters observed in GPGV−/−B plants did not lead to a major B uptake and to the subsequent mitigation of B deficiency symptoms because of the total absence of B in -B nutrient solution. Consequently, the GPGV-related impairment of the B-transporter upregulation did not imply a minor B uptake nor the exacerbation of B deficiency symptom. Conversely, in environmental conditions with different and variable B availability, as in field, viral interference on B-deficiency plant response may impact symptom expression.

### In the Field, GLMD Symptom Expression May Be Associated With a Different Regulation of the Expression of B Transporters

The results of the experiment conducted in environmentally controlled conditions suggested that GPGV does not influence plant B homeostasis *per se*, but it hampers plant responses to already existing B deficiency stress.

In in-field conditions, B availability can be affected by several soil factors including long-term excess rainfall, which reduces the absorption of this nutrient by plants (Goldberg, 1997; Shorrocks, 1997; Yan et al., 2006). The occurrence of disorders, even in cases of ample B supply in the soil, suggests that B deficiency in plants is probably also induced by the rapid growth that results from favorable environmental conditions or high nitrogen fertilizer levels (Brown and Shelp, 1997). Considering that B is characterized by little mobility and thus its availability is essential at all stages of growth, the spring period with its high rainfall and rapid vegetative growth represents a critical situation that can be overcome during the growing season (Brown and Shelp, 1997). This fact is in line with the remission of GLMD symptoms after veraison, with the production of symptomless shoots and leaves from June onward (Bertazzon et al., 2017). Moreover, the susceptibility of B homeostasis to numerous factors, ranging from environmental conditions to physiological plant status, may explain why, within the same vineyards, GLMD symptoms are displayed only by a restricted (and unpredictable) number of GPGV-infected plants.

For a first confirmation of our hypothesis, we conducted some preliminary investigations on 12 field-grown grapevines. In field, B availability can be affected by several factors, changing over time and the space. Given that gene transcriptional regulation occurs within few hours after the onset of stress (Zhou et al., 2015) and, as revealed by the hydroponic experiment, grapevine leaf tissues can be considered a good target for the study of plant response to B bioavailability, the transcript levels of the B transporters were measured in leaf collected immediately after GMLD symptom occurrence. While neither the GPGV variant nor the virus titer seemed to be linked to GLMD symptom expression, the differential regulation of *VvBOR3*, *VvNIP5*, and *VvNIP6* genes between symptomatic and asymptomatic plants hints at the importance of changes in B homeostasis as a trigger for GLMD symptom expression. The small number of plants considered in a single vineyard, the use of a single grapevine variety and the absence of GPGV-free grapevines did not allow a conclusive modeling of the phenomenon. Thus, this preliminary result, together with data collected in hydroponic experiment, call for the necessity to formulate a more comprehensive overview of the grapevine response to GPGV infection, GLMD symptom occurrence and discontinuous B availability.

## Conclusion

Grapevine Leaf Mottling and Deformation is a grapevine disease characterized by unpredictable symptom outbreak. Specific GPGV features can not be excluded to be the unique triggering factor of GLMD symptom expression, since up to now only some of the viral factors possibly involved in plant pathogenesis (García and Pallas, 2015) have been considered (Bianchi et al., 2015; Bertazzon et al., 2017; Saldarelli et al., 2017; Tarquini et al., 2019a, b). Nevertheless, is emerging the idea that plant virus disease symptoms arise from complex dynamic mechanisms of interplays among virus, host factors and environmental conditions (for reviews see Zhang and Sonnewald, 2017; Osterbaan and Fuchs, 2019). Along this line, our investigations exhort the consideration of a new actor in the interaction between GPGV and grapevine plants, i.e. B availability, and of their possible tripartite effect in GMLD symptom occurrence.

## Data Availability Statement

The datasets generated for this study can be found in the MN587102, MN587103, MN587104, MN587105, MN587106, MN587107, MN587108, MN587109, MN587110, MN587111, MN587112, and MN587113.

## Author Contributions

SB, LP, and PE conceived the study and wrote the manuscript. AL set up the hydroponic system and prepared the plant material. SB and LP performed molecular and phylogenetic analysis with the supervision of MM. LP and RM carried out microscopy observations. FF and MF analyzed plant nutrient content.

## Conflict of Interest

The authors declare that the research was conducted in the absence of any commercial or financial relationships that could be construed as a potential conflict of interest.

## Footnotes

1 http://www.ncbi.nlm.nih.gov/BLAST

2 http://bioinfo.ut.ee/primer3-0.4.0/primer3
