# Supplementary material for: With or Without You: Altered Plant Response to Boron-Deficiency in Hydroponically Grown Grapevines Infected by Grapevine Pinot Gris Virus Suggests a Relation Between Grapevine Leaf Mottling and Deformation Symptom Occurrence and Boron Plant Availability
Source: Front Plant Sci. 2020 Mar 3;11:226. doi: 10.3389/fpls.2020.00226 (PMC7062799; doi:10.3389/fpls.2020.00226)
Supplement: Supplementary file 1 [file Data_Sheet_1.pdf]

Supplemental tables

Supplemental table 1. Experiment setting

Grapevine cane	GPGV detection	GPGV sequence	GPGV variant	other viruses[a]	hydroponic experiment	experimental condition[b]	in-field experiment	experimental condition[c]
1	+	fvg30	C	GRSPaV; HSVd; GYSVd-1	x	+B;-B	x	S
2	+	fvg88	C	GRSPaV; HSVd; GYSVd-1	x	+B;-B	x	S
3	+	fvg01	C	GRSPaV; HSVd; GYSVd-1	x	+B;-B	x	A
4	+	fvg52	C	GRSPaV; HSVd; GYSVd-1	x	+B;-B	x	A
5	+	fvg43	A	GRSPaV; HSVd; GYSVd-1	x	+B;-B	x	A
6	+	fvg50	A	GRSPaV; HSVd; GYSVd-1	x	+B;-B	x	A
7	+	fvg00	A	GRSPaV; HSVd; GYSVd-1	x	+B;-B	x	S
8	+	fvg53	A	GRSPaV; HSVd; GYSVd-1	x	+B;-B	x	S
9	+	fvg84	C	GRSPaV; HSVd; GYSVd-1			x	S
10	+	fvg18	B	GRSPaV; HSVd; GYSVd-1			x	A
11	+	fvg29	A	GRSPaV; HSVd; GYSVd-1			x	A
12	+	fvg86	A	GRSPaV; HSVd; GYSVd-1			x	S
13	-		-	GRSPaV; HSVd; GYSVd-1	x	+B;-B		
14	-		-	GRSPaV; HSVd; GYSVd-1	x	+B;-B		
15	-		-	GRSPaV; HSVd; GYSVd-1	x	+B;-B		
16	-		-	GRSPaV; HSVd; GYSVd-1	x	+B;-B		
17	-		-	GRSPaV; HSVd; GYSVd-1	x	+B;-B		
18	-		-	GRSPaV; HSVd; GYSVd-1	x	+B;-B		
19	-		-	GRSPaV; HSVd; GYSVd-1	x	+B;-B		
20	-		-	GRSPaV; HSVd; GYSVd-1	x	+B;-B		

[a] Detection for 12 viruses was carried out (GPGV, GRSPaV, HSVd, GYSVd-1-2, GVA, GVB, GFkV, GLRaV-1-2-3, GFLV, ArMV, GRVFV, GSyV-1)
[b] Each cane collected in field was divided in two cuttings, equally distributed to +B and -B conditions
[c] A= asymptomatic; S= symptomatic

Supplemental table 2. List of BOR proteins used in phylogenetic analysis. Transmembrane domains (TMD) were predicted using the Phobius program (Kall *et al.*, 2004).

Organism	Protein name and reference	NCBI protein number	Protein length (aa)	TMD
Arabidopsis thaliana	AtBOR1 (Takano *et al.*, 2002)	NP_850469.1	704	10
	AtBOR2 (Miwa *et al.*, 2013)	NP_191786.1	703	10
	AtBOR4 (Miwa *et al.*, 2007)	NP_172999.1	683	9
Brassica napus	BnBOR1;1a (Sun *et al.*, 2012)	NP_001302529.1	701	10
	BnBOR1;1c (Sun *et al.*, 2012)	ADF30188.1	701	10
	BnBOR1;2a (Sun *et al.*, 2012)	XP_022574681.1	705	10
	BnBOR1;2c (Sun *et al.*, 2012)	NP_001303160.1	704	10
	BnBOR1;3a (Sun *et al.*, 2012)	ADF30190.1	703	10
	BnBOR1;3c (Sun *et al.*, 2012)	XP_022553077.1	704	10
Citrus macrophylla	CmBOR1 (Canon *et al.*, 2013)	ABQ52428.1	714	10
Hordeum vulgare	xBOT1 (Sutton *et al.*, 2007)	ABS83563.1	666	10
Oryza sativa	OsBOR1 (Nakagawa *et al.*, 2007)	XP_015620545.1	711	10
Vitis vinifera	VvBOR1 (Perez-Castro *et al.*, 2012)	XP_002282501.1	720	12
	VvBOR2	XP_010652294.1	717	10
	VvBOR3	NP_001267820.1	721	10
Zea mays	ZmBOR1 (Chatterjee *et al.*, 2014)	NP_001151747.1	709	12

Supplemental table 3. List of NIP sequences used for the construction of the phylogenetic tree.

Organism	Protein name and reference	NCBI protein number	Protein length (aa)
Arabidopsis thaliana	AtNIP5;1 (Takano *et al.*, 2006)	NP_192776	304
	AtNIP6;1 (Tanaka *et al.*, 2008)	NP_178191.1	305
	AtNIP1;2 (Wang *et al.*, 2017)	NP_193626.1	294
	AtNIP3;1 (Xu *et al.*, 2015)	NP_174472.2	323
Brassica napus	BnaNIP5;1 (Diehn *et al.*, 2019)	XP_013684074.1	301
	BnaNIP6;1 (Diehn *et al.*, 2019)	XP_013727031.1	305
Citrus trifoliate	CiNIP5 (An *et al.*, 2012)	AFN37617.1	300
Oryza sativa	OsNIP2;1 (Ma and Yamaji, 2006)	XP_015626173.1	298
	OsNIP2;2 (Ma and Yamaji, 2006)	XP_015644134.1	298
	OsNIP3;1 (Hanaoka *et al.*, 2014)	AAG13499.1	241
Solanum lycopersicum	SlNIP5;1 (di Gioia *et al.*, 2017)	NP_001274288.1	295
Vitis vinifera	VvNIP5;1	XP_002276319.1	298
	VvNIP6;1	XP_002272988.1	354
Zea mays	ZmNIP3;1 (Leonard *et al.*, 2014)	NP_001105021.1	302
	ZmNIP2;1 (Gu *et al.*, 2012)	NP_001105637.1	295

Supplemental table 4. F-values and P-values of a two-way ANOVA for biometric parameters, such as canopy and root weight, internode lenght and leaf number, evaluating the main and interactive effects of boron (B) availability and virus (GPGV) infection. F and P values in bold are signficant differences at P < 0.05.

**Biometric parameter**	**Test**	**F-value**	**P-value**
Canopy weight	B	**82,684**	**<0,001**
	GPGV	0,243	0,629
	interaction	0,633	0,438
Root weight	B	**38,684**	**<0,001**
	GPGV	0,0181	0,895
	interaction	0,056	0,816
Internode lenght	B	**29,241**	**<0,001**
	GPGV	0,0406	0,843
	interaction	0,407	0,532
Leaf number	B	**46,297**	**<0,001**
	GPGV	0,883	0,361
	interaction	0,0509	0,824

Supplemental table 5. F-values and P-values of a two-way ANOVA for nutrient content in root and leaf evaluating the main and interactive effects of boron (B) availability and virus (GPGV) infection. F and P values in bold are significant differences at P < 0.05.

organ	nutrient	test	F-value	P-value	notes
root	Ca	B	**26,049**	**<0,001**	
		GPGV	3,382	0,086	
		interaction	0,0356	0,853	
	K	B	1,474	0,244	
		GPGV	2,232	0,156	
		interaction	0,0536	0,82	
	Mg	B	**17,371**	**<0,001**	
		GPGV	0,0245	0,878	
		interaction	0,275	0,607	
	P	B	0,337	0,57	
		GPGV	0,263	0,616	
		interaction	0,127	0,727	
	B	B	**248,683**	**<0,001**	
		GPGV	1,965	0,181	
		interaction	1,923	0,186	
	Cu	B	**171,598**	**<0,001**	
		GPGV	4,063	0,062	
		interaction	0,00541	0,942	
	Fe	B	0,000171	0,99	
		GPGV	3,891	0,067	
		interaction	0,258	0,619	
	Mn	B	0,171	0,685	GPGV-/-B
		GPGV	0,324	0,578	≠
		interaction	**10,9**	**0,005**	GPGV+/-B
	Na	B	3,584	0,078	
		GPGV	0,993	0,335	
		interaction	2,773	0,117	
	Zn	B	**31,73**	**<0,001**	
		GPGV	0,142	0,711	
		interaction	2,483	0,136	

organ	nutrient	test	F-value	P-value	notes
leaf	Ca	B	**29,789**	**<0,001**	
		GPGV	1,928	0,182	
		interaction	2,454	0,135	
	K	B	**10,233**	**0,005**	GPGV-/-B
		GPGV	**7,317**	**0,014**	≠
		interaction	**5,997**	**0,025**	GPGV+/-B
	Mg	B	**50,814**	**<0,001**	
		GPGV	1,711	0,207	
		interaction	0,906	0,354	
	P	B	**6,971**	**0,017**	
		GPGV	0,0031	0,956	
		interaction	3,776	0,068	
	B	B	**1277,587**	**<0,001**	
		GPGV	0,51	0,484	
		interaction	4,119	0,057	
	Cu	B	0,0508	0,824	
		GPGV	1,39	0,254	
		interaction	0,686	0,418	
	Fe	B	**38,752**	**<0,001**	
		GPGV	1,355	0,26	
		interaction	0,18	0,677	
	Mn	B	**20,308**	**<0,001**	
		GPGV	1,663	0,214	
		interaction	1,73	0,205	
	Na	B	**82,927**	**<0,001**	GPGV-/-B
		GPGV	**9,356**	**0,007**	≠
		interaction	**10,541**	**0,005**	GPGV+/-B
	Zn	B	**85,768**	**<0,001**	GPGV-/-B
		GPGV	**12,942**	**0,002**	≠
		interaction	1,719	0,206	GPGV+/-B

Supplemental table 6. F-values and P-values of a two-way ANOVA for gene expression in root and leaf evaluating the main and interactive effects of boron (B) availability and virus (GPGV) infection. F and P values in bold are significant differences at P < 0.05.

organ	gene	test	F-value	P-value	notes
root	*VvBOR1*	B	**24,872**	**<0,001**	
		GPGV	0,114	0,74	
		interaction	1,219	0,286	
	VvBOR2	B	**36,159**	**<0,001**	GPGV-/-B
		GPGV	4,113	0,062	≠
		interaction	3,186	0,096	GPGV+/-B
	VvBOR3	B	2,044	0,173	GPGV-/-B
		GPGV	3,423	0,084	≠
		interaction	**6,548**	**0,022**	GPGV+/-B
	VvNIP5	B	**593,936**	**<0,001**	
		GPGV	2,757	0,118	
		interaction	0,0906	0,768	
	VvNIP6	B	**8,552**	**0,01**	
		GPGV	0,032	0,86	
		interaction	0,049	0,828	
leaf	*VvBOR1*	B	**56,621**	**<0,001**	GPGV-/-B
		GPGV	**18,103**	**<0,001**	≠
		interaction	3,707	0,075	GPGV+/-B
	VvBOR2	B	**13,249**	**0,002**	GPGV-/-B
		GPGV	**11,191**	**0,004**	≠
		interaction	**6,321**	**0,023**	GPGV+/-B
	VvBOR3	B	1,001	0,334	
		GPGV	1,738	0,209	
		interaction	0,0616	0,808	
	VvNIP5	B	**56,504**	**<0,001**	GPGV-/-B
		GPGV	4,232	0,059	≠
		interaction	2,177	0,162	GPGV+/-B
	VvNIP6	B	**38,601**	**<0,001**	GPGV-/-B
		GPGV	4,019	0,065	≠
		interaction	3,685	0,076	GPGV+/-B

References

An, J. C., Liu, Y. Z., Yang, C. Q., Zhou, G. F., Wei, Q. J., & Peng, S. A. (2012). Isolation and expression analysis of CiNIP5, a citrus boron transport gene involved in tolerance to boron deficiency. Sci. Hortic. 142, 149-154. doi: 10.1016/j.scienta.2012.05.013

Cañon, P., Aquea, F., Rodríguez-Hoces de la Guardia, A., and Arce-Johnson, P. (2013). Functional characterization of Citrus macrophylla BOR1 as a boron transporter. Physiol. Plant. 149, 329–339. doi: 10.1111/ppl.12037

Chatterjee, M., Tabi, Z., Galli, M., Malcomber, S., Buck, A., Muszynski, M., et al. (2014). The borate efflux transporter ROTTEN EAR is required for maize inflorescence development and fertility. Plant Cell 26, 2962–2977. doi: 10.1105/tpc.114.125963

Di Gioia, F., Aprile, A., Sabella, E., Santamaria, P., Pardossi, A., Miceli, A., et al. (2017). Grafting response to excess boron and expression analysis of genes coding boron transporters in tomato. Plant Biol. 19(5), 728-735. doi: 10.1111/plb.12589

Diehn, T. A., Bienert, M. D., Pommerrenig, B., Liu, Z., Spitzer, C., Bernhardt, N., et al. (2019). Boron demanding tissues of Brassica napus express specific sets of functional Nodulin26 -like Intrinsic [illegible] Plant J. 100(1), 68-82. doi: 10.1111/tpj.14428

Gu, R., Chen, X., Zhou, Y., and Yuan, L. (2012). Isolation and characterization of three maize aquaporin genes, ZmNIP2; 1, ZmNIP2; 4 and ZmTIP4; 4 involved in urea transport. BMB Rep. 45(2), 96-101. doi: 10.5483/BMBRep.2012.45.2.96

Hanaoka, H., Uraguchi, S., Takano, J., Tanaka, M., and Fujiwara, T. (2014). OsNIP3;1, a rice boric acid channel, regulates boron distribution and is essential for growth under boron -deficient conditions. Plan[illegible] 902. doi: 10.1111/tpj.12511

Käll, L., Krogh, A., and Sonnhammer, E. L. (2004). A combined transmembrane topology and signal peptide prediction method. J. Mol. Biol. 338(5), 1027-1036. doi: 10.1016/j.jmb.2004.03.016

Leonard, A., Holloway, B., Guo, M., Rupe, M., Yu, G., Beatty, M., et al. (2014). tassel-less1 encodes a boron channel protein required for inflorescence development in maize. Plant Cell Physiol. 55, 1044–1054. doi: 10.1093/pcp/pcu036

Ma, J. F., and Yamaji, N. (2006). Silicon uptake and accumulation in higher plants. Trends Plant Sci. 11, 392–397. doi: 10.1016/j.tplants.2006.06.007

Miwa, K., Takano, J., Omori, H., Seki, M., Shinozaki, K., and Fujiwara, T. (2007). Plants tolerant of high boron levels. Science 318, 1417. doi: 10.1126/science.1146634

Miwa, K., Wakuta, S., Takada, S., Ide, K., Takano, J., Naito, S., et al. (2013). Roles of BOR2, a boron exporter, in cross linking of rhamnogalacturonan II and root elongation under boron limitation in Arabidopsis. Plant Physiol. 163, 1699–1709. doi: 10.1104/pp.113.225995

Nakagawa, Y., Hanaoka, H., Kobayashi, M., Miyoshi, K., Miwa, K., and Fujiwara, T. (2007). Cell-type specificity of the expression of OsBOR1, a rice efflux boron transporter gene, is regulated in response to boron availability for efficient boron uptake and xylem loading. Plant Cell 19, 2624–2635. doi: 10.1105/tpc.106.049015

Pérez-Castro, R., Kasai, K., Gainza-Cortés, F., Ruiz-Lara, S., Casaretto, J. A., Peña-Cortés, H., et al. (2012). VvBOR1, the grapevine ortholog of AtBOR1, encodes an efflux boron transporter that is differentially expressed throughout reproductive development of Vitis vinifera L. Plant Cell Physiol. 53, 485–494. doi: 10.1093/pcp/pcs001

Sun, J., Shi, L., Zhang, C., and Xu, F. (2012). Cloning and characterization of boron transporters in Brassica napus. Mol. Biol. Rep. 39(2), 1963-1973. doi: 10.1007/s11033-011-0930-z

Sutton, T., Baumann, U., Hayes, J., Collins, N. C., Shi, B. J., Schnurbusch, T., et al. (2007). Boron-toxicity tolerance in barley arising from efflux transporter amplification. Science 318, 1446–1449. doi: 10.1126/science.1146853

Takano, J., Noguchi, K., Yasumori, M., Kobayashi, M., Gajdos, Z., Miwa, K., et al. (2002). Arabidopsis boron transporter for xylem loading. Nature 420, 337–340. doi: 10.1038/nature01139

Tanaka, M., Wallace, I. S., Takano, J., Roberts, D. M., and Fujiwara, T. (2008). NIP6;1 is a boric acid channel for preferential transport of boron to growing short tissues in Arabidopsis. Plant Cell 20, 2860–2875. doi: 10.1105/tpc.108.058628

Wang, Y., Li, R., Li, D., Jia, X., Zhou, D., Li, J., et al. (2017) NIP1; 2 is a plasma membrane-localized transporter mediating aluminum uptake, translocation, and tolerance in Arabidopsis. Proc. Natl. Acad. Sci. USA 114, 5047-5052. doi: 10.1073/pnas.1618557114.

Xu, W., Dai, W., Yan, H., Li, S., Shen, H., Chen, Y., et al. (2015). Arabidopsis NIP3;1 plays an important role in arsenic uptake and Root-to-Shoot translocation under arsenite stress conditions. Mol. Plant 8, 722–733. doi: 10.1016/j.molp.2015.01.005
